# Coral Skeleton δ^15^N as a Tracer of Historic Nutrient Loading to a Coral Reef in Maui, Hawaii

**DOI:** 10.1038/s41598-019-42013-3

**Published:** 2019-04-03

**Authors:** Joseph Murray, Nancy G. Prouty, Sara Peek, Adina Paytan

**Affiliations:** 1 Ocean Sciences Department, UC Santa Cruz, 1156 High Street, Santa Cruz, California 95064 United States; 2 U.S.G.S., Pacific Coastal and Marine Science Center, 2885 Mission Street, Santa Cruz, California 95060 United States; 3 U.S.G.S., 345 Middlefield Road, Menlo Park, California 94025 United States; 4 Institute of Marine Sciences, UC Santa Cruz, 1156 High Street, Santa Cruz, California 95064 United States

## Abstract

Excess nutrient loading to nearshore environments has been linked to declining water quality and ecosystem health. Macro-algal blooms, eutrophication, and reduction in coral cover have been observed in West Maui, Hawaii, and linked to nutrient inputs from coastal submarine groundwater seeps. Here, we present a forty-year record of nitrogen isotopes (δ^15^N) of intra-crystalline coral skeletal organic matter in three coral cores collected at this site and evaluate the record in terms of changes in nitrogen sources. Our results show a dramatic increase in coral δ^15^N values after 1995, corresponding with the implementation of biological nutrient removal at the nearby Lahaina Wastewater Reclamation Facility (LWRF). High δ^15^N values are known to be strongly indicative of denitrification and sewage effluent, corroborating a previously suggested link between local wastewater injection and degradation of the reef environment. This record demonstrates the power of coral skeletal δ^15^N as a tool for evaluating nutrient dynamics within coral reef environments.

## Introduction

Coral reefs are facing increasing stress from global climate change (e.g., increasing temperatures, ocean acidification, sea-level rise) combined with local stresses from over-fishing, sedimentation, and terrestrial sources of pollution^1,2^. Specifically, declining water quality in the coastal environment, related to factors such as upstream changes in catchment basin land-use (e.g., deforestation, agriculture), enhanced terrestrial runoff ^3^, submarine groundwater discharge (SGD)^4,5^ and coastal urbanization^6^, among others, is believed to be an important cause of coral reef decline^7^. Increased inputs of terrestrial nutrients can impact the structure of marine biotic communities and may lead to eutrophication, harmful algal blooms^8^, decreased coral abundance and diversity^9^, and increased macro-algal abundance^3,10^. In particular, excess inputs of nitrogen (N) and phosphorous can alter ecosystem function and structure by shifting reefs dominated by corals to algae dominance^11,12^, and increase vulnerability of reefs to coral disease^13,14^. High nutrient loading when coupled with low pH has also been shown to increase the sensitivity of corals to bioerosion^15^. Nutrient inputs to coral reefs can originate from a variety of different sources, and it is vital to identify specific sources in order to design and evaluate effective mitigation strategies. Coral skeleton geochemistry, particularly δ^15^N (δ^15^N = [[(^15^N:^14^N_sample_)/(^15^N:^14^N_reference_)] − 1] × 1000), provides one method for understanding changes in historic nutrient loading^16–19^.

δ^15^N measurements are commonly used to distinguish between different N sources^20^. Specifically, dissolved inorganic nitrogen (DIN) derived from sewage typically has δ^15^N values ranging from +7‰ to +38‰, with values as high as 74‰ occurring at our study site^20,21^. This is due to a variety of processes that occur during sewage treatment, including bacterial denitrification of nitrate, nitrification of ammonia or ammonium, and ammonia volatilization. The net result of these processes is the consumption of nitrate, leaving the residual DIN pool heavily enriched in ^15^N^20^. δ^15^N of synthetic N fertilizer, which is often derived from atmospheric N_2_, ranges between −4‰ and +4‰^22,23^. The δ^15^N of marine nitrate, which is controlled by a complex balance of local and global biogeochemical processes, is typically between +4‰ and +7‰, with a deep ocean average value of approximately +5.5‰^24^. However, euphotic zone δ^15^N values can be higher due to uptake and utilization by phytoplankton^25^.

δ^15^N values have been used in a variety of different systems to investigate the impact of anthropogenic N fluxes. This includes tracing inputs of nitrate from septic tanks into groundwater^26^ and tracing runoff of fertilizer into various ecosystems^27^. Dissolved nitrate isotopes (δ^15^N and δ^18^O) have also been used to evaluate mixing of different anthropogenic nitrate sources in coastal and estuarine systems^20,28,29^. Organic matter δ^15^N has been applied for tracing N inputs to soils^30^, sediments^31^, fish^32^, crustaceans^33^, algae^34,35^, and food-webs^36^. Similarly, the δ^15^N of organic matter contained within scleractinian coral skeletons has been shown to directly relate to sources of N for corals^18,37,38^. As such, records of coral organic matter δ^15^N have been used to determine sources of N and changes in nutrient dynamics over various timescales^17,18,37,39–45^.

**Figure 1:**
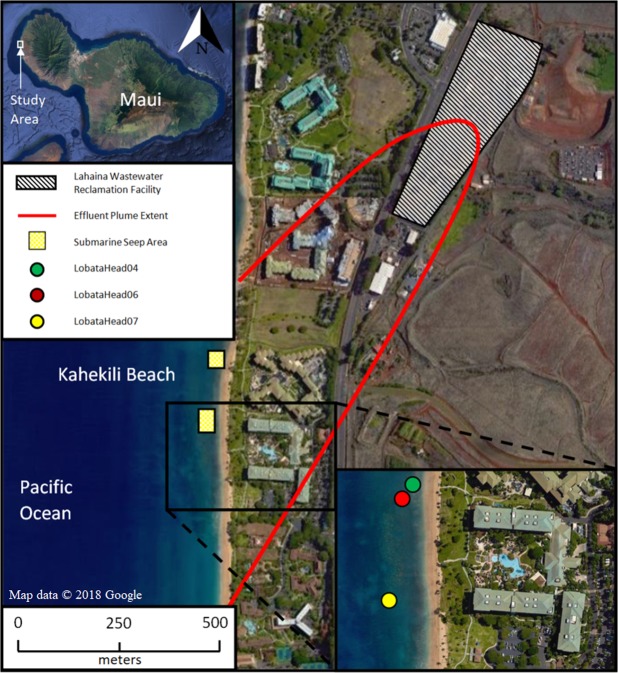
Kahakili Beach Park (KBP) and Lahaina Wastewater Reclamation Facility. Overview of the study site, showing the KBP coastline, LWRF, and surrounding area on the west shore of Maui, Hawaii. The red line shows the extent of the injection plume within the coastal aquifer as mapped by dye tracer experiments^54^. Cores LobataHead04 and LobataHead06 were collected within an active seeps area, while Core LobataHead07 was collected ~150 m to the south, away from the seeps (see inset). Imagery 2018 Google, map data 2018 Google.

Here we use a new method (see methods below) to measure intra-crystalline δ^15^N in coral cores collected off the west coast of Maui, Hawaii in order to shed light on changes in N sources to the local reef system as they relate to coral reef health. Adjacent to a densely inhabited shoreline with known input from land-based sources of pollution, the reef at Kahekili Beach Park (KBP), in North Kannapali, Maui, Hawaii (Fig. 1) has shown signs of degradation since state monitoring began in 1995^46–48^, including large, periodic macro-algal blooms^49^ and up to 40% loss of coral coverage between 1995 and 2005, with specific areas showing nearly 100% coral loss (termed “dead zones”)^46^. These “dead zones,” which are highly patchy and heterogeneously distributed throughout the reef, are characterized by a local high abundance of dead coral skeletons and represent areas of historical coral mortality^46,47^. Results of a study in 2009 suggested that coral cover and degradation at this site are associated with local fresh groundwater seeps that have been suspected as conduits for sewage effluent injected into the aquifer at the LWRF^46,50^. The discharging water is typically nutrient rich, and previous studies at this location employing hydrological models and δ^15^N in algae suggest a terrestrial N source characterized by denitrification, possibly sewage effluent^35,50–54^. Glenn *et al*. (2012; 2013)^53,54^ used dye tracers along with natural geochemical tracers and aerial thermal infrared imagery to confirm a direct hydrological link between the LWRF and the small submarine seeps discharging along the reef. However, it is possible that other sources of N downstream from the LWRF, or natural processes within the aquifer, are driving the elevated N concentrations and δ^15^N values. Specifically, there is no instrumental record of water quality or N content available for this site prior to the early 1990’s so it is hard to determine if the N loading and associated coral decline can be categorically attributed to the LWRF treated sewage injection. Therefore, an independent measure directly linking N input at the seeps and the sewage treatment facility (i.e. a “smoking gun”) in both a modern and historical context is needed.

Land-use in West Maui has historically been dominated by both sugar cane and pineapple cultivation. From the early 1900’s until 1979, land-use practices in this area remained relatively unchanged, with limited urban development along the coastline. Beginning in 1979, large scale plantation agriculture began to decline, with the area experiencing a 21% decline in agricultural land-use and a corresponding 43% increase in urban land use between 1979 and 2004^55^. Much of this decline in agricultural area can be attributed to the closing of a major sugar cane plantation in 1999. Pineapple cultivation also began to decrease in the late 1990’s before stopping entirely in 2009^56^. Much of this formerly agricultural land remains fallow today, with some sections converted to either diversified agriculture or urbanized areas. Currently, much of the coastline consists of commercial development, resorts, and golf courses, with former agricultural land and state forests on the slopes above.

The LWRF is located approximately three miles north of the town of Lahaina in the Kaanapali District of West Maui. The facility is responsible for treating all municipal sewage for the local community, which can swell to over 40,000 people during the tourist season. The facility generates a primary stream of tertiary treated wastewater, which is subsequently disposed on-site via four injection wells, along with a secondary stream of tertiary treated wastewater that is additionally disinfected using UV radiation to produce water that meets R-1 standards for reuse in local landscaping. Tertiary treatment consists of primary settling to remove suspended solids, secondary aerobic biodegradation by microbial metabolism to remove organic matter, and biological nutrient removal in order to remove DIN. The first plant at the LWRF was constructed in 1976 and is capable of an average flow capacity of about 3.2 million gallons per day (mgd). A second plant, with a capacity of 6.7 mgd, was constructed in 1985 and has now largely replaced the first plant for day-to-day operation. This second plant was upgraded in 1995 in order to incorporate biological nutrient removal as well as a partial UV disinfection system^57^. Due to an Environmental Protection Agency mandate, chlorination disinfection techniques were used from October 2011 until May 2014, after which full UV disinfection was operational. Detailed records of treated wastewater injection rates over the lifetime of the facility are not readily available, however today the plant processes an average of ~4 mgd of raw municipal sewage from the broader Lahaina area^58^.

Here we use δ^15^N of intra-crystalline coral skeletal organic matter in *Porites lobata* coral cores collected from three locations, representing sites adjacent to seep discharge and far from the direct seep influence, along the fringing reef at KBP to construct a record of δ^15^N in the water at each of the three sites. The KBP is located along the west shore of Maui, Hawaii ~0.5 km southwest of the LWRF (Fig. 1, see Glenn *et al*. (2013) for detailed site description^54^). Two of the cores collected (LobataHead04 and LobataHead06) were from within an active seep area^52–54^, whereas core LobataHead07 was collected ~150 m to the south, in an area of high coral cover and low bioerosion and background nutrient concentrations^59^. Intra-crystalline organic matter from each core was analyzed for δ^15^N variability using a newly developed analytical procedure (see methods). In this study we present a record of historic changes in N sources to the coast in relation to changes in land-use and sewage treatment practices upstream of KBP over the last several decades.

## Results and Discussion

**Figure 2:**
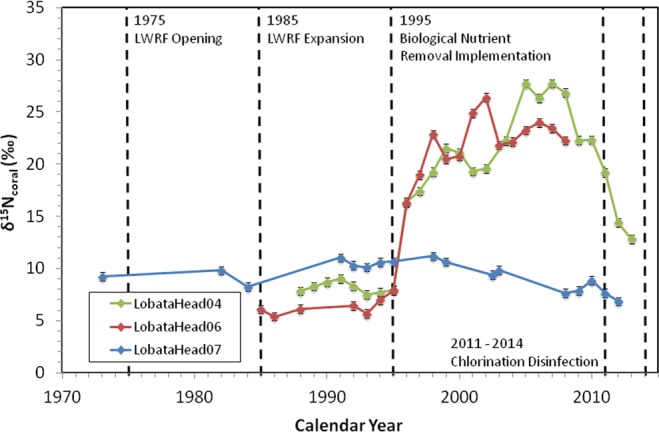
Historic record of coral skeletal δ^15^N. Coral skeletal δ^15^N values plotted through time. Major milestones in the LWRF operation are also noted. Cores LobataHead04 and LobataHead06 both show a sharp increase in δ^15^N between 1995 and 1996 in response to the implementation of biological nutrient removal at the LWRF, while core LobataHead07 does not show any response to LWRF activities.

The coral skeletal δ^15^N values measured in cores collected adjacent to the SGD seeps (LobataHead04 and LobataHead06) show distinct trends compared to the coral collected outside the seep area (LobataHead07, Fig. 2). The largest differences are seen after 1995, with corals collected adjacent to the SGD seeps consistently showing δ^15^N values greater than +10‰, and as high as +27.7‰, while coral δ^15^N values from outside the seep area remain relatively constant, centered at ~+10‰. Prior to 1995, coral δ^15^N values from all three sites are more similar to each other, varying between +6 and +10‰.

Beginning in 1996, coral δ^15^N values adjacent to the SGD seeps increase rapidly, from less than +8‰ to over +16‰ in one year. Biological nutrient removal, consisting primarily of heterotrophic denitrification, was implemented at the LWRF beginning in 1995 to treat the wastewater prior to injection. This N removal process has been an integral part of the wastewater treatment facility since 1995. N removal continues within the anoxic groundwater plume downstream of the injection wells^58^. As a result, a large amount of N is removed prior to discharge at the SGD seeps, leaving behind DIN with an enriched δ^15^N fingerprint that discharges into the coastal zone at the submarine seeps. Upon discharge the groundwater with ^15^N enriched DIN mixes to various degrees with seawater which has DIN with lower δ^15^N values. This mixed, DIN pool which is still enriched in ^15^N relative to surrounding seawater is available for uptake by the corals and is recorded in the skeletal organic matter. The average aquifer transfer time between the injection wells and the SGD seeps, as determined through dye tracer experiments, is approximately 14 months^53,54^. Thus, the high δ^15^N coral signature observed starting in 1996 is most likely a direct response to the biological nutrient removal process implemented the previous year, providing a direct link between the nutrient-enriched SGD and the LWRF. If natural processes, such as denitrification along the flow path in the aquifer were the source of the high δ^15^N values and associated nutrient loads at the seeps, such values would have been seen prior to the start of biological nutrient removal process implementation at the LWRF.

Coral δ^15^N values from cores collected adjacent to the seeps continue to increase for the next decade, peaking between 2002 and 2007, and reaching a maximum of +27.7‰ in 2007 (LobataHead04) and +26.4‰ around 2002 (LobataHead06). Note that LobataHead06 was collected dead so there is some uncertainty as to the exact age model (see methods). However, coral δ^15^N values do not rise monotonically, instead displaying year-to-year variability, especially between 1998 and 2002. A direct comparison of δ^15^N values between the two corals collected at the seeps shows that while the overall trends are similar, the detailed patterns for these two coral heads are not identical. For example, coral δ^15^N values decrease slightly between 1999 and 2002 before increasing again in 2003 in LobataHead04, whereas coral δ^15^N values in LobataHead06 less than 16 m away increase sharply during the same time period, before decreasing again in 2003. This δ^15^N variability between LobataHead04 and LobataHead06 is likely due to changes in SGD flow rate, local hydrodynamics, and the degree of dilution with seawater at the individual coral collection site. Prouty *et al*.^21,59^ analyzed seep water and nearby seawater chemistry at this site during a 6-day intensive sampling campaign. The results of these studies revealed that bottom water carbonate chemistry, nutrient concentrations, and specifically nitrate δ^15^N were largely controlled by SGD discharge fluxes, as well as nearshore oceanographic conditions (e.g. wave height), and varied significantly over the sampling period. For example, nitrate concentrations within the seep discharge fluid varied from 0.45 µmol L^−1^ to over 70 µmol L^−1^, while nitrate δ^15^N values varied from less than +40‰ to almost +75‰, all during the 6-day period, demonstrating the highly dynamic nature of this reef location. Higher nitrate concentrations are observed at the shallowest sampling sites closest to the seep^21,59^ consistent with previous work at this site^52–54^. Collectively these data suggest that the two corals (LobataHead04 and LobataHead06) likely experienced somewhat different conditions both day-to-day and at any specific time. This could explain the variability observed in δ^15^N in each coral and the differences between these corals from 1998 to 2002. The variability in coral δ^15^N values is also consistent with other δ^15^N measurements from the study area, including those in particulate organic matter (POM) (this study, see Table 1), groundwater and seawater DIN^53,54,59^, and algal tissue^35,51^, capturing how differences in exposure to nutrient-enriched effluent and post-discharge mixing can lead to differences in δ^15^N. Despite the high variability in nitrate δ^15^N, the coral skeleton provides a time-integrated signal, meaning the overall signal is robust. Importantly, the δ^15^N variability between LobataHead04 and LobataHead06 is also much smaller than the primary denitrification signal observed after 1996.

**Table 1:** Physical characteristics of coral cores and chemical characteristics of the surrounding seawater.

Core ID	LobataHead04	LobataHead06	LobataHead07
Location	SGD Seep	SGD Seep	Southern Drill Site
Latitude	20° 56.326′ N	20° 56.318′ N	20° 56.236′ N
Longitude	156° 41.587′ W	156° 41.589′ W	156° 41.611′ W
Distance From Seep (m)	16	0	156
Water Depth (m)	<2	<2	3
Collection Status	Alive	Dead	Alive
Isotope Date Range Sampled	1988–2013	1983–2008	1973–2013
Bioerosion (% vol)*	5.9	14.6	n/a
Growth Rate (cm yr^−1^)*	0.72	0.69	0.9
Seawater Nitrate (µmol L^−1^)*	0.41 ± 0.18 (n = 37)	20.35 ± 23.32 (n = 37)	n/a
POM δ^15^N (‰)	+6.6 ± 2.1 (n = 17)		+3.4 ± 2.2 (n = 9)
2013 Seawater δ^15^N_NO3_ (‰)	+31.5 ± 2.1 (n = 3)		+8.0 ± 1.2 (n = 3)
2016 Seawater δ^15^N_NO3_ (‰)**	+ 70.4 ± 6.5 (n = 28)		n/a

The above table contains a summary of the important physical and chemical characteristics of the coral cores and seawater surrounding each site. Bioerosion, growth rate and nitrate concentration data (indicated by *) were previously published in Prouty *et al*.^21^. 2016 seawater δ^15^N_NO3_ values (indicated by **) were previously published in Prouty *et al*.^59^. Values are reported as average ± standard deviation. Seawater nitrate concentrations and 2016 seawater δ^15^N_NO3_ values based on 6 day sampling period in March 2016, while 2013 seawater δ^15^N_NO3_ was measured on samples collected in December 2013.

In contrast to the high coral δ^15^N values observed after 1995 in the two cores collected adjacent to the SGD seeps, the coral core (LobataHead07) collected offshore in a low nutrient setting does not display an increase in δ^15^N. The isotopic composition of this core remains relatively stable around +10.5‰ prior to 1999, after which there is a gradual decrease to +6.9‰. The lack of any response to the implementation of biological nutrient removal at the LWRF is strong evidence that this area, located approximately 150 m south of the active SGD seeps, is outside the direct influence of the SGD-derived wastewater effluent, given both the low nutrient concentrations and high percentage of coral cover observed^59^. This core is therefore primarily reflecting trends in the DIN isotopic composition of coastal seawater and reflecting regional changes in background groundwater (including degree of denitrification within the aquifer and input from other sources) and seawater DIN δ^15^N not affected by the LWRF effluent. In addition, coral δ^15^N signatures may be influenced by coral feeding behavior. For example, corals can obtain nutrients from seawater directly^60,61^ when concentrations are high (as they are at the seeps), whereas that coral δ^15^N signal can be muted by heterotrophic feeding on a dynamically mixed N pool, particularly when DIN concentrations are lower (as they are at LobataHead07). The increased development, urbanization (24.5% increase in population between 2000 and 2010)^46^ and fertilizer use in golf courses in the region over time likely contribute to the decreasing δ^15^N trend seen in LobataHead07 from 1999 to 2012. These N sources typically have δ^15^N ratios lower than offshore seawater^62^.

The coral skeletal δ^15^N compositions of all cores show a decreasing trend by the mid-2000’s, with the trends being more dramatic in the two cores collected adjacent to the seep. We note that while the above described δ^15^N increases can only be due to denitrification, the more recent δ^15^N decreases may be explained by several other factors. The decreasing trend and lower values could be the result of changes in LWRF injection rates, decreases in the efficiency of the biological nutrient removal processes, or some other factor operating within the aquifer. However, since the coral skeleton records the combined result of these processes, it is difficult to identify the relative importance of each process. The ~3‰ decrease observed in LobataHead07 (southern core away from the seeps) over this time period suggests that a small portion of the overall decreasing trend in LobataHead04 and LobataHead06 may not be directly related to effluent injection at the LWRF, and instead related to more regional processes, such as increased urbanization and tourism as discussed above. However, the much larger (>10‰) decrease in the two cores adjacent to the seeps indicates the contribution of additional processes unique to the SGD influence. As previously mentioned, the LWRF effluent was subjected to chlorination disinfection prior to injection from October 2011 to May 2014, when UV disinfection was fully implemented. Chlorination disinfection may be responsible for suppressing microbial activity including that responsible for N removal^58^. This in turn would result in a decrease in the overall rate of denitrification, and thus an increase in rate of DIN discharge and a decrease in δ^15^N in the effluent DIN. Indeed, within 14 months of chlorination beginning at the LWRF, increased nutrient loads from SGD were observed on the ref.^58^, and at the same time LobataHead04 shows a decrease in δ^15^N from +19.2‰ to +12.8‰ between 2011 and 2013. Chlorination disinfection was discontinued in 2014 hence a rebound in δ^15^N would be expected after 2014. This rebound cannot be captured in our record since the coral cores were collected in 2013. However, this rebound in δ^15^N has been observed in dissolved nitrate at the site. In December 2013, the average seawater nitrate δ^15^N value at the seeps was +31.6‰ (this study, n = 3, Table 1) but in 2016 this value was over +70‰ (n = 28, Table 1)^59^. Both values are higher than those recorded in the coral skeleton likely because the corals do not derive their N directly from DIN and may include N from heterotrophy. However, as noted previously coral δ^15^N values in LobataHead04 start decreasing two years prior to the onset of chlorination, indicating that other factors in addition to the chlorination treatment have contributed to the overall trend. The exact N sources or processes that contribute to the decrease in δ^15^N, other than those associated with the LWRF, are hard to pinpoint but could be related to the large increase (~20%) in tourists visiting Maui and related increased contribution from fertilizer use in resorts and golf course landscaping^62^. Following the US financial crisis of 2007–2008, Maui’s economy turned around and entered a period of economic expansion in 2009, leading to the development of new beach resorts and an expansion of the Ka’anapali golf courses^63^.

The skeletal δ^15^N values for all 3 corals prior to 1995 are lower than those following the initiation of biological N removal at the LWRF but are slightly different across the three cores analyzed for this study (Fig. 2). Particularly LobataHead07 which is located far from the SGD seeps records higher δ^15^N values then the corals closer to the seeps. These different δ^15^N values represent differences in the δ^15^N signature of the dominant nutrient sources available to the corals at each location. Coral skeletal δ^15^N values of LobataHead07, which is located south of the primary seep site where nutrient levels are low (<0.1 μmol L^−1^ nitrate^21^) are ~+10‰, are likely influenced by the natural surface seawater nitrate value, which is slightly enriched in ^15^N relative to average deep ocean seawater due to nitrate assimilation (dissolved nitrate in seawater at that site measured in December 2013 was +8‰, Table 1). In contrast, coral δ^15^N values prior to 1995 for cores collected adjacent to the seep are lower than at the southern site, indicating exposure to one or more additional nutrient sources with lower δ^15^N values. At that time, the discharging water at the seeps contained N from a mixture of fresh groundwater and sewage-derived injection fluids that were not subject to denitrification at the treatment facility. This effluent-impacted groundwater is mixed to various degrees with local seawater prior to being incorporated into the coral. Hence the δ^15^N of these corals prior to 1995 (between ~+6‰ and +8‰) reflect the sewage-derived N source prior to implementation of denitrification at the LWRF operation, and hence less impacted by denitrification, and N in the local groundwater. The corals collected at the seep site do not represent the period of skeletal growth prior to LWRF operation (i.e. prior to 1975). Therefore, it is difficult to determine the relative contribution of inputs of sewage-effluent and those of groundwater or other sources (such as potential inputs from agricultural runoff containing fertilizer) to the δ^15^N of the corals prior to 1995. However, if we assume a value of 10‰ (as seen in LobataHead07) is representative of background conditions in the region at that time, then the sewage effluent must have contributed substantial amounts of N to reduce the signature by 2–4‰, although the exact contribution cannot be determined without knowing the isotopic signature of the effluent itself.

**Figure 3:**
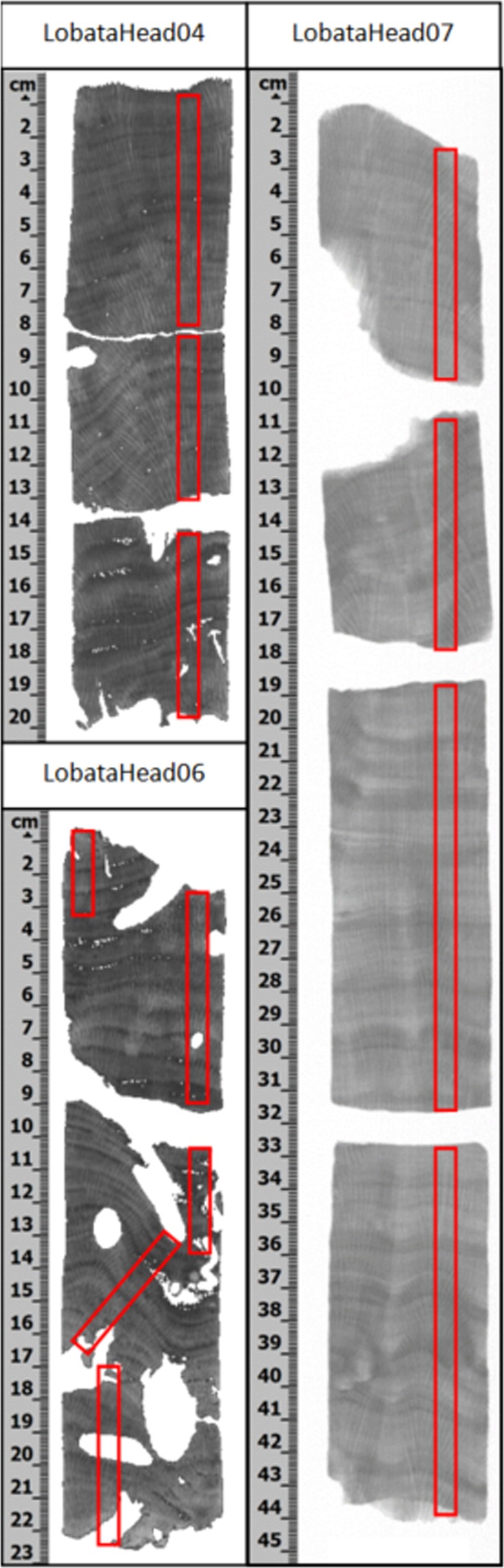
Coral core imagery. CT scan imagery of cores LobataHead04 and LobataHead06 (left), and X-Ray imagery of core LobataHead07 (right). The areas labeled in red represent the areas on the cores that were subsampled for δ^15^N analysis parallel to the coral growth axis, with a sample track width of ~0.5 cm on average.

Cores collected adjacent to the seep display a variety of physical signs suggesting they have been exposed to multiple SGD-associated stressors (Table 1 and Fig. 3), including nitrate concentrations up to 50 times higher than ambient seawater, and lower pH bottom water^21^. For example, Prouty *et al*.^21^ found lower calcification rates and increased bioerosion in corals collected adjacent to the seeps; bioerosion rates were much higher than those observed in coral cores collected in the Pacific under equivalent low pH conditions but living in oligotrophic waters^15^. Bioerosion rate and percent bioerosion volume were significantly positively correlated (p ≤ 0.05) with the surrounding seawater nitrate concentration (Table 1) suggesting that eutrophication exacerbates ocean acidification and bioerosion of corals^21^.

Our results confirm that corals living within the SGD seep area are impacted by sewage-effluent injected at the LWRF. The fact that the δ^15^N signature of the corals collected from within the seep area increases dramatically within one year after the implementation of biological nutrient removal at the LWRF validates a direct link between wastewater injection and water quality on the reef, and is strong evidence that wastewater effluent, and its associated nutrient loads, is a dominant nutrient source to this location of the reef. More broadly, our results show that δ^15^N of coral intra-skeletal organic matter is useful for understanding changes in nutrient sources to coral reefs over time. Many of the most threatened reefs around the world are severely lacking in historic data that is crucial for making informed management decisions. The information provided by coral geochemical records as presented here can help close these important knowledge gaps and inform efforts to reduce nutrient transport to the nearshore environment.

## Methods

### Field Sampling

Coral cores from both alive and dead *Porites lobata* coral heads were collected in July of 2013 as part of an ongoing U.S. Geological Survey study aimed at quantifying the impact of SGD to the fringing refs.^21,59^. Coral cores from two shallow water (<5 m) locations in close vicinity to actively discharging seeps and another ~150 m to the south, away from active seeps, collected using a hand-held, air-powered pneumatic drill were used for this study. Cores are 50 mm in diameter, and range in length from 15 to 55 cm. In addition to the coral cores, samples of local seawater and discharging vent water were collected for nutrient and nitrate isotope analysis as reported in Prouty *et al*.^21^ and summarized in Table 1. Seawater samples were also collected and filtered for particulate organic matter (POM) in October 2013.

### Coral Chronologies

Two of the coral cores from KBP used here were obtained from living *Porites lobata* coral heads (LobataHead04 and LobataHead07). This allowed for the development of chronologies based on visually counting the annual growth bands detected using X-ray and CT imaging, counting backwards from the date of collection (assuming each band represents one year). For the core collected from a dead specimen (LobataHead06), radiocarbon (^14^C) analysis was used to estimate the age of the coral (at the National Ocean Sciences Accelerator Mass Spectrometry, Woods Hole). ^14^C values were measured at five depths within the core and compared to bomb-derived ^14^C values previously measured in Hawaii, which determined that the coral died in approximately 2008^64^. This established the age of the upper-most growth layer, with the rest of the chronology being developed by visually counting the annual growth bands in the X-ray imagery. Due to the relatively shallow slope of the recent radiocarbon bomb-curve, there is a relative uncertainty of several years for the age of the skeleton of LobataHead06. However, the agreement in the timing of the sharp increase in δ^15^N between LobataHead04 and LobataHead06 and their close physical proximity suggests that the chronology should be reasonably well constrained although small offsets of a few years are possible.

### Coral Subsampling and Preparation

The coral cores were sliced in half along the major growth axis to ensure sampling along the major growth axis. Using the X-ray and CT images as a guide, annually resolved coral subsamples were collected by grinding down into the coral to a depth of approximately 3 mm, using a handheld Dremel tool, following the growth banding laterally, to produce ~100 mg of coral fine powder. The coral slabs were cleaned using a compressed air stream between each sample to avoid cross-contamination.

### Isolation of Coral Intra-crystalline Organic Matter

In order to remove any external organic N that is subject to diagenesis and may not always preserve the original δ^15^N^45^, a bleach oxidation/extraction was employed, following part of the method described in Wang *et al*.^65^. Powdered coral samples were transferred into 10 mL acid-cleaned plastic centrifuge tubes with 5 mL of a 10 wt% NaClO bleach solution (10–15% available chlorine). Samples were then capped, shaken, and placed on an orbital shaker table for 24 hours. After 24 hours on the shaker table, samples were centrifuged, the solution was decanted, and the remaining powder was rinsed with 5 mL of deionized water. This rinse step was repeated 3 times before the samples were centrifuged, water decanted, and powder left to dry in an oven at 40 °C overnight. The cleaned powder samples were then stored inside a desiccator until isotope analysis of the intra-crystalline organic matter. The efficacy of this cleaning method was rigorously tested using an in-house carbonate standard material (described below) in order to ensure the efficient and effective removal of any external organic matter.

### δ^15^N Isotopic Analysis – Nano-EA-IRMS

Due to the extremely low intra-crystalline organic N content in aragonitic corals, large amounts of coral skeletal material are necessary for isotope analysis. In order to reduce the amount of material required and allow for a sufficiently high temporal resolution, thereby increasing our ability to detect rapid changes, a modified Elemental Analyzer-Isotope Ratio Mass Spectrometer (EA-IRMS) “nanoEA” was developed following the work of Polissar *et al*.^66^. Due to the very high C:N ratio of bulk coral skeletal material, our modified design utilizes two cryogenic traps that separate N_2_ from CO_2_ and vent the CO_2_ to the atmosphere. The N_2_ gas is then concentrated by cryotrapping and transferred to the mass spectrometer. This allows for the introduction of a small, pure N_2_ sample to the mass spectrometer for determining both weight % N and δ^15^N. An advantage to this approach is that the procedure does not require the use of denitrifying bacteria for the production of sample gas, and therefore can be used at facilities that do not maintain such cultures.

Supplementary Fig. S1 (see Supplementary Information) shows a schematic diagram of the modified elemental analyzer and gas valve design that enables this separation technique. First, the samples are placed into a “zero-blank,” air-sealed autosampler that is purged with helium (He) carrier gas in order to eliminate any atmospheric N_2_ contamination. Next, the samples are dropped and combusted in a standard elemental analyzer that is in-line with the gas valve and IRMS system, with the combustion column at 1030 °C, the reduction column at 650 °C, and a He carrier gas flow pressure of 150 kPa. The CO_2_ and N_2_ gas that is produced in the EA then flows towards the two-way valve, and subsequently passes first through a CO_2_ trap, and then through an N_2_ trap. Each trap is submerged in liquid N_2_ and is designed to concentrate and separate the two gases from one another. We used a relatively large-diameter coiled gas line as the CO_2_ trap to accumulate the large volume of CO_2_ generated by these carbonate samples. The N_2_ trap consists of coiled molecular-sieve tubing that restricts the flow of N_2_ at liquid N_2_ temperatures, but will release the flow at higher temperatures. Once all of the sample gas has flowed from the EA and through the two traps, the position of the two-way value is changed, changing the source and direction of the gas flow through the traps and valve. The two traps are then raised in sequence, first allowing the sample N_2_ gas to flow back through the valve and towards the IRMS, and then allowing the collected CO_2_ to vent through the valve into the atmosphere. Rigorous testing of the valve, system timings, flow characteristics and IRMS settings using our in-house carbonate standard material (see below) was conducted to ensure that large quantities of carbonate are fully combusted, and that CO_2_ is completely separated from the N_2_ of interest, and that δ^15^N values obtained are accurate. Specifically, the trap and valve timings were optimized in order to ensure complete sample collection while minimizing the contribution of any background N_2_ in the system.

Unknown sample weights ranged between 60 and 80 mg of processed coral powder, depending on the % N content of the material, with the goal of introducing approximately 6 µg of N per sample. In order to monitor for system leaks and any potential reduction in combustion efficiency or sample carryover, all samples and standards were bracketed by blanks during each run. An internal consistency standard consisting of homogenized coral skeleton material was produced early on in the method development process. This material was used to assess the efficacy of the bleach cleaning as well as the efficiency of the carbonate combustion described above, but it was also used as an in-run and between-run internal standard. Numerous samples of this material were analyzed interspersed throughout each instrument run in order to monitor and correct for instrument drift, and in order to better compare sample results from run to run. While this material has not been independently standardized using a separate analytical method, we have run the material hundreds of times during method development, validation and sample analysis, and use the consistency of the measured value in order to evaluate the accuracy and stability of the analytical method. The across-run average δ^15^N value of this material as measured during the analysis of these corals was + 5.79‰ ± 0.31‰ (n = 25, see Supplementary Fig. S2 in Supplementary Materials) based on calibration using certified standards (see below), with typical intra-run precision below 0.5‰.

All data were corrected for both size and isotopic value using a suite of three independently calibrated, homogenized internal lab standards (EDTA, δ^15^N = 0.72‰; spirulina, δ^15^N = 10.88‰; fish fertilizer, δ^15^N = 16.24‰) as well as one calibrated international isotope standard (IAEA-N-2, δ^15^N = 20.3‰). Due to the very small amount of N being introduced to the mass spectrometer for each sample, accurately characterizing and correcting for the contribution of the blank is extremely important. In order to do this, we run a size range series of each of the above organic standard materials during each instrument run, generally ranging from 2 µg to 8 µg of N. The measured relationship between peak area and sample weight is used to calculate the mass of N present in each sample. Finally, the measured non-linear relationship between standard size and isotopic value is used to do a two-point isotope value calibration for each unknown sample. δ^15^N precision for replicate sample analysis is approximately 0.4‰ based on sample replicates, as well as repeated internal consistency standard coral analyses. The approximate size of the instrument blank, as calculated following Polissar *et al*.^66^, typically ranged from 500 to 900 ng N. The size and isotopic value of the blank was somewhat variable run-to-run, however this was rigorously characterized using the above analysis scheme during each individual run and was typically stable within a run after purging the autosampler.

## Supplementary information

Supplementary Materials
