# Supplementary material for: Coral Skeleton δ15N as a Tracer of Historic Nutrient Loading to a Coral Reef in Maui, Hawaii
Source: Sci Rep. 2019 Apr 3;9:5579. doi: 10.1038/s41598-019-42013-3 (PMC6447536; doi:10.1038/s41598-019-42013-3)
Supplement: Supplementary file 1 — Supplementary Materials [file 41598_2019_42013_MOESM1_ESM.pdf]

Coral Skeleton $\delta^{15}N$ as a Tracer of Historic Nutrient Loading to a Coral Reef in Maui, Hawaii

Joseph Murray†, Nancy G. Prouty§, Sara Peek ∥, Adina Paytan‡*

† Ocean Sciences Department, UC Santa Cruz, 1156 High Street, Santa Cruz, California 95064, United States, jmurray1@ucsc.edu

§ USGS, Pacific Coastal and Marine Science Center, 2885 Mission Street, Santa Cruz, California 95060, United States

∥ USGS, 345 Middlefield Road, Menlo Park, California 94025, United States

‡ Institute of Marine Sciences, UC Santa Cruz, 1156 High Street, Santa Cruz, California 95064, United States

* Corresponding author – apaytan@ucsc.edu

Supplementary Materials

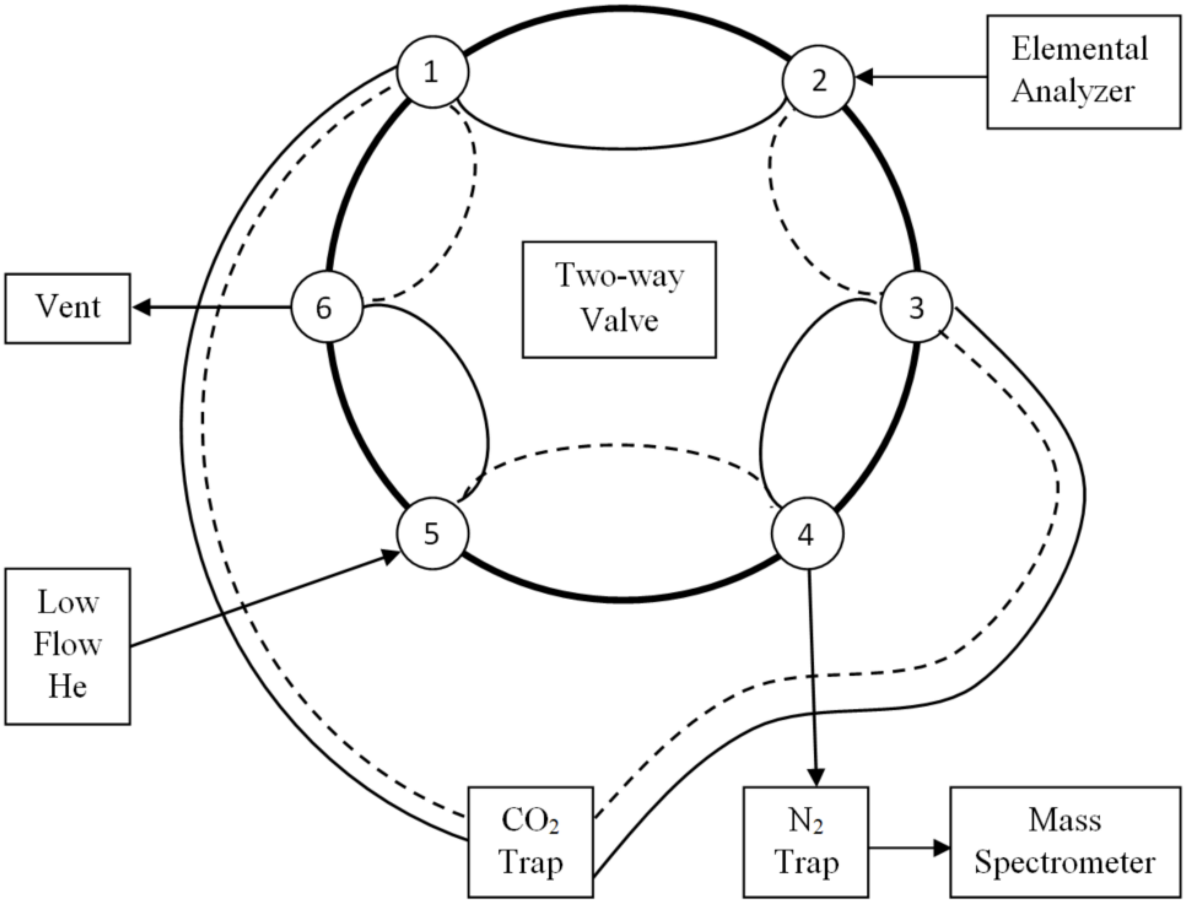

Supplemental Figure S1: NanoEA instrumentation schematic

Schematic diagram of the two-way gas valve and cryotrap setup for the nanoEA instrumentation. The solid lines represent sample gas pathways with the valve in the starting position during sample combustion, trapping and separation. The dashed lines represent sample gas pathways with the value in the open position, venting the carbonate-derived CO_2 to the atmosphere while injecting the N_2 sample gas of interest into the mass spectrometer using a low-flow He carrier gas.

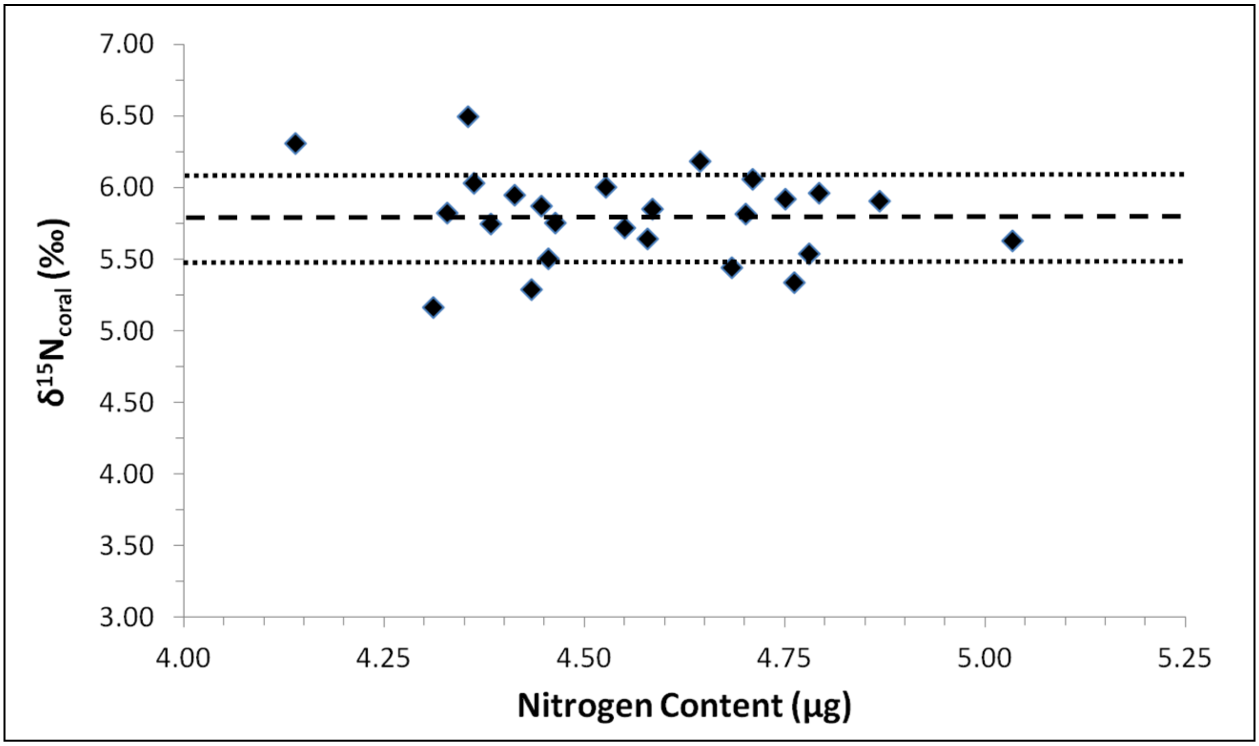

Supplemental Figure S2: Long term internal standard results

Measured internal standard carbonate $\delta^{15}N$ values plotted against nitrogen content (µg N as determined from peak area) for all internal standard samples analyzed during data acquisition for the coral samples described in this manuscript. The dashed lines represent the average value ± one standard deviation across all coral sample analysis (+5.79‰ ± 0.31‰).
